# Conceptualizing Indigenous strengths-based health and wellness research using group concept mapping

**DOI:** 10.1186/s13690-023-01066-7

**Published:** 2023-04-26

**Authors:** Victoria M. O’Keefe, Tara L. Maudrie, Ashley B. Cole, Jessica S. Ullrich, Jillian Fish, Kyle X. Hill, Lauren A. White, Nicole Redvers, Valarie Blue Bird Jernigan, Jordan P. Lewis, Amy E. West, Charlene Aqpik Apok, Evan J. White, Jerreed D. Ivanich, Katie Schultz, Melissa E. Lewis, Michelle C. Sarche, Miigis B. Gonzalez, Myra Parker, Sophie E. Neuner Weinstein, Celena J. McCray, Donald Warne, Jessica C. Black, Jennifer R. Richards, Melissa L. Walls

**Affiliations:** 1 Center for Indigenous Health, Johns Hopkins Bloomberg School of Public Health, Baltimore, MD USA; 2 Department of Psychology, Oklahoma State University, Stillwater, OK USA; 3 IREACH, University of Washington, Seattle, WA USA; 4 Minneapolis VA Health Care System, Center for Care Delivery and Outcomes Research, Minneapolis, MN USA; 5 Department of Indigenous Health, University of North Dakota School of Medicine & Health Sciences, Grand Forks, ND USA; 6 Joint Program for Social Work and Psychology, University of Michigan, Ann Arbor, MI USA; 7 Schulich School of Medicine & Dentistry, University of Western Ontario, London, ON Canada; 8 Center for Indigenous Health Research and Policy, Oklahoma State University, Tulsa, OK USA; 9 Memory Keepers Medical Discovery Team, University of Minnesota Medical School, Duluth, MN USA; 10 Keck School of Medicine, Children’s Hospital Los Angeles, Department of Pediatrics, University of Southern California, Los Angeles, CA USA; 11 Cloudberry Consulting, Anchorage, AK USA; 12 Laureate Institute for Brain Research, Tulsa, OK USA; 13 University of Colorado Anschutz Medical Campus, Centers for American Indian and Alaska Native Health, Aurora, CO USA; 14 University of Michigan School of Social Work, Ann Arbor, MI USA; 15 Department of Family and Community Medicine, University of Missouri School of Medicine, Columbia, MO USA; 16 School of Medicine, Department of Psychiatry and Behavioral Sciences, University of Washington, Seattle, WA USA; 17 Department of Obstetrics and Gynecology, Oregon Health and Sciences University, Portland, OR USA; 18 Northwest Portland Area Indian Health Board, Portland, OR USA; 19 Department of Alaska Native Studies and Rural Development, University of Alaska Fairbanks, Fairbanks, AK USA; 20 Center for Indigenous Health--Great Lakes Hub, Johns Hopkins Bloomberg School of Public Health, Duluth, MN USA

**Keywords:** American Indian/Alaska Native, Indigenous, Public health, Group concept mapping, Conceptual framework

## Abstract

**Background:**

In recent years public health research has shifted to more strengths or asset-based approaches to health research but there is little understanding of what this concept means to Indigenous researchers. Therefore our purpose was to define an Indigenous strengths-based approach to health and well-being research.

**Methods:**

Using Group Concept Mapping, Indigenous health researchers (*N* = 27) participated in three-phases. Phase 1: Participants provided 218 unique responses to the focus prompt “Indigenous Strengths-Based Health and Wellness Research…” Redundancies and irrelevant statements were removed using content analysis, resulting in a final set of 94 statements. Phase 2: Participants sorted statements into groupings and named these groupings. Participants rated each statement based on importance using a 4-point scale. Hierarchical cluster analysis was used to create clusters based on how statements were grouped by participants. Phase 3: Two virtual meetings were held to share and invite researchers to collaboratively interpret results.

**Results:**

A six-cluster map representing the meaning of Indigenous strengths-based health and wellness research was created. Results of mean rating analysis showed all six clusters were rated on average as moderately important.

**Conclusions:**

The definition of Indigenous strengths-based health research, created through collaboration with leading AI/AN health researchers, centers Indigenous knowledges and cultures while shifting the research narrative from one of illness to one of flourishing and relationality. This framework offers actionable steps to researchers, public health practitioners, funders, and institutions to promote relational, strengths-based research that has the potential to promote Indigenous health and wellness at individual, family, community, and population levels.

## Background

Indigenous communities and scholars have long prioritized and advocated for strengths-based approaches to public health and wellness research, interventions, funding, and policy. This advocacy movement is occurring in parallel to increasing national agendas emphasizing Indigenous leadership to alter societal narratives and the creation of Indigenous futures aligned with cultural values [1]. The emphasis on strengths-based approaches often desired by Indigenous communities is challenging in that agencies supporting community programming and health interventions often require risk-focused outcomes not reflective of Indigenous values and approaches to health and wellness [2, 3]. Risk-focused outcomes are instead more aligned with deficits-based models that draw from Eurocentric definitions of health that may be harmful to Indigenous communities in terms of stigmatization and perpetuation of negative stereotypes that Indigenous Peoples are “prone to ill health and in need of intervention” [2–4]. In sum, if the focus is solely on pathology, pathology is what will be found.

There continues to be calls for a shift in gaze from individual level determinants of health to broader system determinants of Indigenous Peoples health including oppressive histories, policies, and practices that remain unaddressed including that of colonization [5–7]. Although relevant and important to address, Indigenous communities also carry strengths, wellness, and wisdom to resist, survive, heal, and persist despite the systemic pressures to disconnect from culture, spirit, and wholistic collectivism [8, 9]. These strengths and assets within Indigenous Nations are well delineated at the community level; however, a definition or framework for an “Indigenous strengths-based approach to health and wellness research” specifically has yet to be clearly translated. We take steps to address this gap in existing public health literature by engaging Indigenous scholars in a collaborative process for brainstorming, rating, and describing strengths-based Indigenous health research.

## Eurocentric strengths-based approaches

A focus on strengths in the Western context has roots in Eurocentric psychology and social work; however, these Western-based approaches have been critiqued by Indigenous scholars. Positive psychology suggests that basic, individual-level principles that allow people to flourish apply to all humans globally [10]. In contrast to the *Diagnostic and Statistical Manual of Mental Disorders* that identifies pathology, the *Character Strengths and Virtues: A Handbook and Classification* identifies globally recognized character strengths and virtues that make individuals happy, strong, and resilient [11]. While this classification system references global work across historical eras to identify positive virtues, it has been critiqued for excluding cultural contexts across ethnic/racial minoritized groups [12].

Strengths-based approaches centering resilience were developed in the field of social work as a client-centered philosophy of practice to emphasize clients’ skills, attitudes, and cognitions in the face of adversity to drive individual behavior change. These approaches grew as alternative responses to past dominant practice approaches, which centered “expert” clinicians, their diagnoses, and prescriptive approaches as drivers of clients’ positive health behavior change [13, 14]. Strengths-based approaches that rely on individual thriving and resilience are an increasingly common philosophy across a variety of social work, research, and health practice settings [3]. In an adjacent vein, social-ecological approaches understand strengths and barriers to health promotion as sourced in the social environment [15]. These approaches conceptualize distinctive levels of strength-based factors: individual, interpersonal, organizational, and societal, which are commonly implemented in public health research and practice [16].

Most of these approaches (i.e., positive psychology, resilience, social-ecological model) fall short in conceptualizing and understanding Indigenous strengths due to their emphasis on individualism and the lack of attention to social, historical, and cultural contexts. Indigenous scholars argue that both resilience and social-ecological approaches are largely informed by Eurocentric concepts of individuality which often ultimately emphasize deficits or are rooted in response to adversity [3, 5, 8, 17, 18]. In contrast, Indigenous knowledges suggest that health promotion, well-being, and flourishing exists within and beyond the individual [19]. For instance, socio-cultural approaches emphasize multi-level and community attributes. Thus, socio-cultural approaches are aligned more closely with Indigenous concepts of resilience that understand strengths as deriving from social relations, collective identities, and traditional practices despite ongoing experiences of discrimination and structural violence [20, 21].

## Indigenous voices and leadership: current project

It is crucial that Indigenous voices, concepts, and paradigms are brought to the forefront of health and wellness research. Indigenous strengths-based research is “founded upon Indigenous knowledges and guided by Indigenous people” [22]. Indigenous strengths-based research sustains cultural knowledges *and* advances research agendas that are for and by Indigenous Peoples (e.g., “Nothing about Indigenous peoples, without Indigenous peoples”) [23]. Therefore, to prevent epistemic injustices like those caused by colonial research, knowledge co-created with Indigenous communities should encourage local learning, local action, and should directly benefit Indigenous Peoples and communities [24].

## Methods

Group Concept Mapping (GCM) is a method for gathering group consensus on a topic and developing conceptual frameworks [25]. GCM uses participatory methods to gather qualitative and quantitative data and analyzes these mixed-methods data using multidimensional scaling and hierarchical cluster analyses. GCM occurs in three phases: 1) idea generation; 2) data organizing; and 3) interpretation. In idea generation, each participant is asked to provide as many responses as they would like to a focus prompt. In the data organization phase, participants are asked to group statements and to rate statements on scales identified a priori by the study team. In the final phase, participants are brought together to interpret maps resulting from analyses and to create cluster names.

There is currently limited literature on Indigenous conceptual frameworks, which are vital to guide research with Indigenous communities [22]. Creating a framework to guide strengths-based public health research is a transformative step in building a renewed approach to research with Indigenous communities and scholars. Therefore, the aim of this project was to engage a collective of Indigenous health scholars in Group Concept Mapping (GCM) to define and inform a conceptual framework on an Indigenous strengths-based approach to health and wellness research.

### GCM facilitation team

Three Indigenous scholars (Authors VMO, TLM, and MLW) planned the study design and methodology. Within this process, each team member centered their Tribal values and teachings within research planning meetings. They discussed the importance of relationality, community-based values, honoring others through providing a gift, and expressing gratitude. The team agreed to provide an honorarium and co-authorship to scholars who joined the project to honor their time and knowledge. During interpretation meetings, the GCM facilitation team opened with expressing their deep gratitude to scholars who participated and led an activity with a door prize, reflective of community gatherings that team members attend and participate in.

### Participants

We utilized a purposive sampling frame [26] to invite Indigenous scholars whose work emphasizes community strengths. Thirty-seven Indigenous scholars were invited to participate. A total of *N* = *27* Indigenous scholars participated in at least one of three phases: *n* = 24 participated in phase 1, *n* = 26 participated in phase 2, and *n* = 22 participated in phase 3. Scholars reported working in Indigenous health and wellness research from 2 to 46 years (*M* = 13.0). There was a wide range of self-reported areas of research, and scholars could select more than one research area: mental health (31%), substance use (21%), traditional medicine/healing (13%), environmental health (9%), food systems/nutrition (6%), diabetes/obesity (6%), reproductive health (4%), infectious disease (4%), gender identities (3%) and education (3%). Nearly half reported their research is conducted with rural or reservation communities (46%), 27% reported their research takes place with urban/suburban communities, and 27% reported a combination or working with urban and rural/reservation communities.

#### Phase 1: Idea generation

Each scholar was asked to brainstorm responses to the focus prompt: “*Indigenous Strengths-Based Health and Wellness Research…*” They were instructed to provide as many responses as possible to this prompt within a 28-day period. Twenty-four Indigenous scholars provided 218 responses. Following idea generation, the GCM facilitation team engaged in a process of idea synthesis to ensure that each statement included one unique idea, was relevant to the prompt, was clear and understandable, as well as to reduce redundancies and provide a reasonable number of statements that participants could sort and rate in Phase 2 [27]. Authors VMO and TLM worked independently to reduce and edit the statement set, then met to discuss discrepancies, and finally, the GCM facilitation team (Authors VMO, TLM, and MLW) met via a hybrid meeting (one team member virtual; two in-person) to reach consensus on the final statement set. The idea synthesis process resulted in 94 statements.

#### Phase 2: Data organizing

Each scholar was invited to participate in an unstructured sorting method to organize the ideas generated in Phase 1 into as many categories that make sense to them (i.e., no predetermined number of groupings were provided) [27]. Indigenous scholars were then instructed to sort the 94 statements into groupings that made sense to them and to name these groupings. Each scholar was also asked to rate each statement based on its importance to Indigenous strengths-based health and wellness research using a 4-point Likert-type scale ranging from 1 (*relatively unimportant*) to 4 (*extremely important*). In total 26 participants completed this phase over a 60-day period.

After checking data for completeness, the GCM facilitation team used groupwisdom software [28] to explore and analyze participant sorting and rating data. Using a multi-dimensional scaling algorithm in groupwisdom [28], a point map was created to plot points and show how they were sorted in relation to other statements. In a point map, items that are not frequently sorted together are plotted farther from one another, while items frequently sorted together are plotted closer together. A stress value, a commonly used goodness of fit indicator that assesses the relationship between participants’ groupings of statements and the map generated through multi-dimensional scaling, was used to judge the model’s internal validity [29, 30]. The stress value of our multi-dimensional scaling was 0.311. This value falls within the 95% confidence interval for GCM studies established by Trochim in 1993 [31]. A systematic review of GCM studies found an average stress value of 0.28 with a range of 0.17 to 0.34 [30]. Additionally, our stress value of 0.311 falls below the established upper limit of 0.39 (meaning that for studies whose stress values fall below 0.39 there is a less than 1% chance of random arrangement of objects or no structure underlying arrangement) [32]. Based on this evidence, the model presented has reasonable internal validity.

**Fig. 1:**
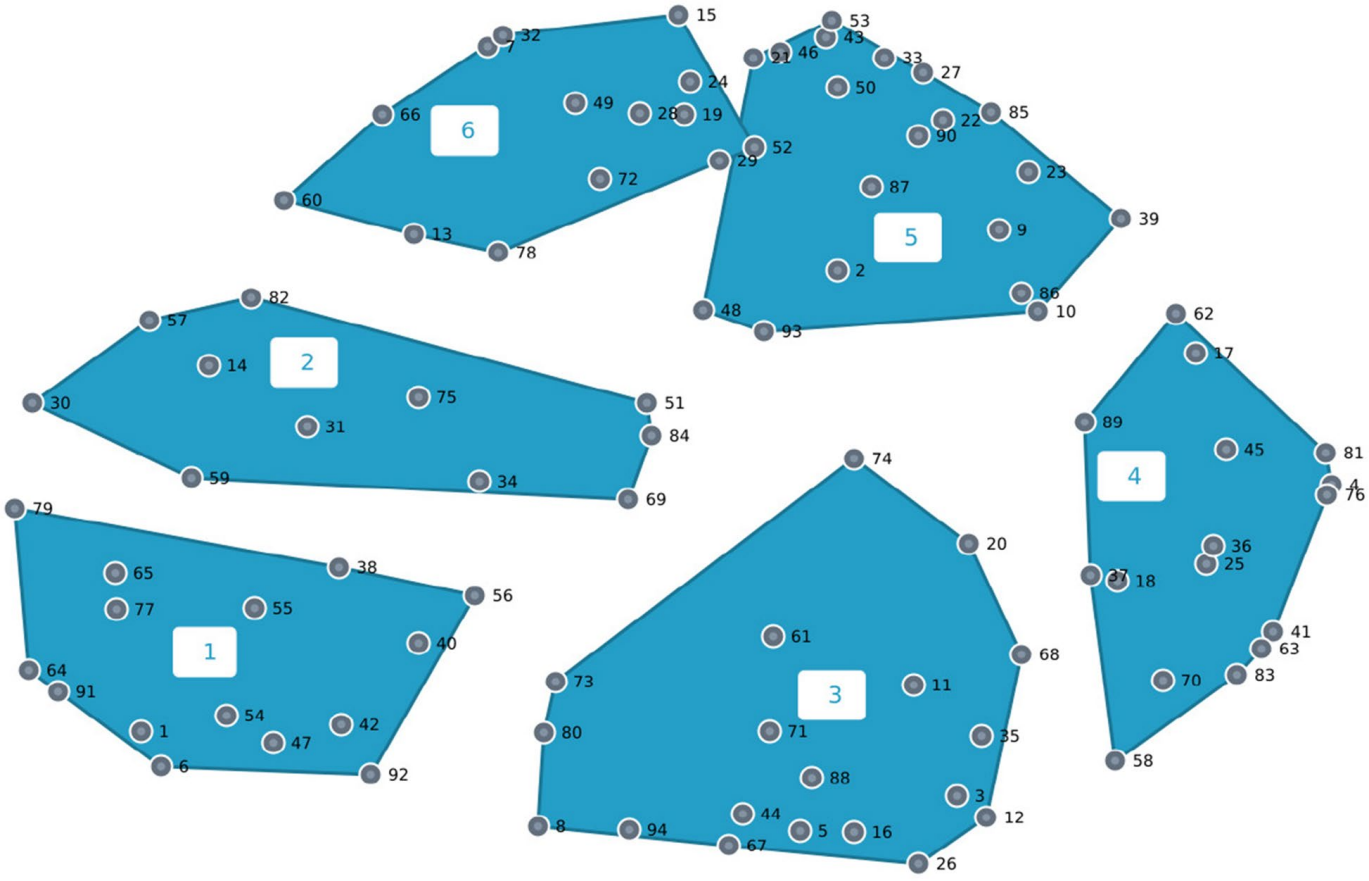
Cluster map using hierarchical cluster analysis

Following creation of the point map, hierarchical cluster analysis was performed using Ward’s Method to group statements that are closest to one another into groups called clusters. Ward’s method creates a structure resulting from data similarities used for interpretation [33] and results in several potential cluster solutions. The GCM facilitation team (VMO, TLM, MLW) independently reviewed several cluster solutions and reached consensus for a six-cluster solution which appeared to best fit the data (See Fig. 1). The importance rating data was used to create a cluster rating map that displays on average how each statement in the cluster is rated on a scale of 1 to 4 (1 = *relatively unimportant* to 4 = *extremely important*). As these analyses were grounded in data that were entirely participant generated, the resulting cluster map reflects how the whole group sorted and rated statements that represent Indigenous strengths-based health and wellness research.

#### Phase 3: Interpretation

For the final phase, two virtual synchronous meetings were held via a video conferencing software, with all scholars from Phases 1 and 2 invited to participate. A total of *N* = 22 Indigenous scholars attended Phase 3 meetings (*n* = 13 in the first meeting “group one”; *n* = 9 in the second meeting; “group 2”). During meetings, scholars were presented with phase 1 and 2 results and asked to share their interpretations of the point and cluster maps. They were also asked to consider each cluster and the statements it contained, and to propose names for each cluster. In each meeting, robust conversations occurred verbally and in written form in the chat function. These conversations informed the final names of each cluster. One GCM facilitation team member reviewed transcripts from both virtual meetings and combined feedback to name and describe each cluster (see Results below).

#### IRB approval

This project was determined to be non-human subjects research by the Johns Hopkins Bloomberg School of Public Health Institutional Review Board (IRB: IRB00017142).

## Results

The 94 brainstormed statements (Table 1) were sorted into a concept map with six clusters (Fig. 1). Below we provide a description of each cluster using Indigenous researchers’ interpretation that occurred in two group meetings and derive tangible ways these interpretations translate to action steps to promote Indigenous strengths-based health research (Table 2).

**Table 1:** Final statements by cluster

Cluster number	Cluster title	Statements
1	Decolonial Research: Exists, Resists, Persists in the Face of Colonialism	**1.** Should be prioritized by all research funding sources **6.** Should be recognized through formal academic policies and procedures **38.** Is anticolonial **40.** Is surviving and thriving in the face of colonization and its effects **42.** Exists, resists, and persists despite colonialism **47.** Requires Indigenous and non-Indigenous researchers to think critically about how they benefit from colonial structures **54.** Requires decolonizing methodologies that are not currently supported by health and academic systems **55.** Recognizes that data is not a thing to be extracted and owned by outsiders **56.** Highlights that no person's experience is statistically insignificant **64.** Can be met with skepticism because it is research **65.** Is championed by many Native researchers and activists **77.** Thrives within healthy research environments **79.** Requires time that funding mechanisms do not often afford **91.** Can be difficult to accomplish in U.S. academic institutions **92.** Seeks to find new statistical and research methodologies that help to discover how culturally-grounded programs help Native people
2	Indigenous Praxis: Positionality and Process for Being in Good Relation	**14.** Should be done by people with authentic care, concern, and love for Indigenous communities **30.** Acknowledges cumulative trauma since colonization **31.** Needs to be done thoughtfully **34.** Focuses on well-being rather than disorder **51.** Are honest in their approach to benefit and uplift Indigenous communities, their cultures, languages, and knowledges **57.** Takes patience **59.** Uses many methods **69.** Is multi-level **75.** Is contextual **82.** Is co-designed with Indigenous peoples **84.** Supports and grows Indigenous pedagogies
3	Generating and Transforming Indigenous Futures	**3.** Should be used to help shape laws and policies that affect Indigenous communities **5.** Can be narrative shifting (e.g., counters stereotypes) **8.** Builds skillsets, training, and opportunities for growing Indigenous scholars **11.** Defines what health and wellness is **12.** Can teach the world **16.** Should lead to identifying structural changes **20.** Is revolutionary in that it is ancient and iterative **26.** Will educate **35.** Is justice **44.** Leads to liberation from the confines of colonialism **61.** Is innovative **67.** Can turn the gaze from "deficits" of Indigenous peoples to deficits of Western capitalism **68.** Is essential **71.** Can lead the field and provide a framework for broader person and community-centered health and wellness research **73.** Highlights best practices **74.** Will evolve **80.** Can push research as an enterprise towards a resilience and resistance over disparities **88.** Clarifies social determinants of health specific to Indigenous populations **94.** Should build capacity so that future efforts are Indigenous led
4	Intergenerational Healing and Flourishing	**4.** Is healing for Indigenous peoples and communities **17.** Is what communities have been asking for a long time **18.** Is preventative **25.** Will change the health of Native communities for the better **36.** Is restorative **37.** Is transformative **41.** Is generative for Indigenous peoples and our futures **45.** Is empowering for Indigenous peoples and communities **58.** Celebrates Indigenous success **62.** Germinates in Indigenous community conversations **63.** Is about building a life worth living **70.** Is focused on maximizing flourishing **76.** Builds community **81.** Can improve health and well-being for communities around the world **83.** Allows for human potentiality **89.** Always benefits the people and community who provide the knowledge
5	Collective Wisdom: Original Instructions and Dynamic Futures	**2.** Centers Tribal and community sovereignty **9.** Is family and community focused **10.** Is led by Indigenous peoples and communities **21.** Lives within us **22.** Helps reclaim traditional culture and spirituality **23.** Preserves collective wisdom **27.** Honors Mother Earth **33.** Restores traditional forms of living, being, relating, and creating **39.** Places the power of healing in the hands of Indigenous peoples and communities **43.** Means going back to our (Indigenous peoples) roots **46.** Privileges land-based knowledge that is often hyper-local **48.** Imagines Indigenous futures **50.** Is grounded in the teachings and lessons of our Elders **53.** Encourages the use of Indigenous foodways **85.** Upholds intergenerational transmission of knowledge **86.** Centers the priorities of Indigenous peoples **87.** Privileges local understandings and ways of knowing **90.** Includes knowledge and wisdom from youth/future generations **93.** Recognizes the importance of cultural identity in health outcomes
6	Centering Indigenous Ways and Cultures in Research	**7.** Should feel like ceremony **13.** Should be driven by the heart **15.** Accounts for interrelationships of physical, mental, and spiritual health **19.** Is intergenerational **24.** Considers the interests of our ancestors **28.** Is connected to the past **29.** Honors Indigenous ways of knowing **32.** Can be supported with ceremony **49.** Is storytelling **52.** Privileges Indigenous knowledge and traditional ecological knowledge **60.** Invites Indigenous people to exist in all of their pluralities **66.** Operates through relationships which center reciprocity and accountability **72.** Finds approaches to address health disparities grounded in cultural values and practices **78.** Is Indigenized

**Table 2:** Recommendations for Indigenous strengths-based health research

Cluster	Actionable Recommendations
1. Decolonial Research: Exists, Resists, Persists in the Face of Colonialism	• Support Indigenous scholars who are navigating Eurocentric systems (e.g., funding agencies) and their own tribal values • Funding agencies and institutions support strengths-based health research topics • Funding agencies and institutions allocate adequate time and funding to support relational, formative aspects of strengths-based research
2: Indigenous Praxis: Positionality and Process for Being in Good Relation	• Emphasize and act to support relationality, including, but not limited to relationships to community, ancestors, lands, cultures • Promote relational processes of research, including honoring and respecting community relationships • Name, discuss, and address positionality and power within research
3: Generating and Transforming Indigenous Futures	• Research should lead to action (e.g., narrative shifting, advocacy, services, policy, capacity building)
4: Intergenerational Healing and Flourishing	• Research should benefit communities • Report and uplift community action and change through research
5: Collective Wisdom: Original Instructions and Dynamic Futures	• Promote and act according to original (Indigenous) instructions and/or cultural teachings/values • Emphasize intergenerational connectedness • Acknowledge that communities and cultures adapt and evolve over time (e.g., be flexible)
6: Centering Indigenous Ways and Cultures in Research	• Promote Indigenous Research Methodologies • Respect, cite, and employ Indigenous epistemologies, knowledges, cultures, spirituality

### Cluster 1: Decolonial research: exists, resists, persists in the face of colonialism

Scholars in group one shared that this cluster appeared to be about the ongoing necessity of “undoing Western structures” through decolonizing. They agreed that the phrase “exists, resists, persists” observed in statement 42 was powerful and resonated to explain what is happening in Indigenous led health research. Furthermore, they discussed the complexity of Eurocentric structures and Indigenous research. One scholar shared that this cluster appeared to be about “navigating the Western colonial research structures as Indigenous scientists who are hoping to do strengths-based research.” Another scholar described this as “walking in two worlds.” The group reached consensus with the proposed cluster name “Decolonial Research: Exists, Resists, Persists” with the recognition that other clusters may help create balance by focusing on Indigenizing. Group two discussed the word “decolonial” with some raising concerns that “decolonizing” has become a buzz word and is overused, losing its power despite its undeniable importance. Like group one, scholars in group two shared that there is an intersection of Eurocentric colonial structures (e.g., research funding) and Indigenous research. While group two suggested using Indigenous or Indigenist to replace “decolonial” in the title, they also discussed how Indigenous/Indigenist research can stand on its own and is not an approach in response or reaction to colonialism.

### Cluster 2: Indigenous praxis: positionality and process for being in good relation

Both groups agreed that this cluster captured the “who” and the “how” when it comes to Indigenous strengths-focused health research. Group one discussed the process of research that includes “community-centered processes,” honoring and respecting community relationships, and positionality. There was agreement among several scholars about how this cluster related to the concept of “in a good way.” One scholar shared this cluster appeared to be about “all the things we do for the love of community.” Group one shared that this cluster also included statements relating to healing and wellness, the importance of context, and Indigenous praxis. Like group one, scholars in group two agreed that this cluster included statements focused on relationality and the importance of relationships (the “who”), as well as the term praxis (the “how”). There was discussion about the term “relational” and questions regarding in relation to whom or what. For example, group members shared that relationality could include community relationships, but can also include relationships to ancestors, lands, and more.

### Cluster 3: Generating and transforming Indigenous futures

The two groups agreed that this cluster conveyed how Indigenous health and wellness research is transformative. A group two participant stated, “it’s not enough to just do research, it has to do something” and Indigenous health research changes the narrative, advocates, and changes the norm. Both groups agreed that this cluster appeared to be about aspirational goals when working in this transformative way and can lead to beneficial outcomes. Finally, there was consensus across both groups that Indigenous health research can teach others. A participant in group one shared “it’s exemplary – other people [non-Indigenous researchers] should be learning from us rather than the other way around.”

### Cluster 4: Intergenerational healing and flourishing

There was strong agreement across both groups that statements in this cluster focused on intergenerational healing, “thrivance”, and flourishing at the community level. Further, the groups agreed that this cluster appeared to capture community outcomes, community-driven change making, and ensuring that communities benefit from research. One participant shared this cluster “centers beloved community.” There was also agreement across the two groups that there was significant overlap between Cluster 3 and 4. However, there was consensus that Cluster 3 appeared to be more about goals of Indigenous Peoples in the academy or other institutions, whereas Cluster 4 seemed to be focused on Indigenous communities and families.

### Cluster 5: Collective wisdom: original instructions and dynamic futures

There was agreement between both groups that this cluster involved original instructions, (i.e., relational responsibilities to one another, to our environment, our spiritual world, and generations who have come before us, and those that will come after us), [34] and a deep Indigenous focus of knowing one’s roots and stories. A participant in group one shared that the statement “lives within us,” highlighted the internal light and guidance when doing this work. Participants in both groups noted the importance of intergenerational connectedness captured in this cluster and the importance of “knowing the past and imagining the future.” Further, group two underscored that Indigenous Peoples and cultures have and continue to evolve over time, which they felt should be captured in the cluster name and description.

### Cluster 6: Centering Indigenous Ways and Cultures in Research

Both groups discussed how this cluster included statements that focused on two areas: (a) Indigenous epistemologies, knowledges, cultures, spirituality, and transcendence; and (b) Indigenous Research Methodologies. One scholar in the first group shared that this cluster “feels like the ‘Indigenized’ balance to the earlier ‘decolonizing’ one [Cluster 1].” A few scholars in group one discussed that the content in Cluster 6 seemed to overlap with Cluster 5. One participant stated that Cluster 5 seemed to be about guidance and Cluster 6 includes examples of protocols or actions to follow such guidance and instructions.

### Mean rating analysis of clusters

**Fig. 2:**
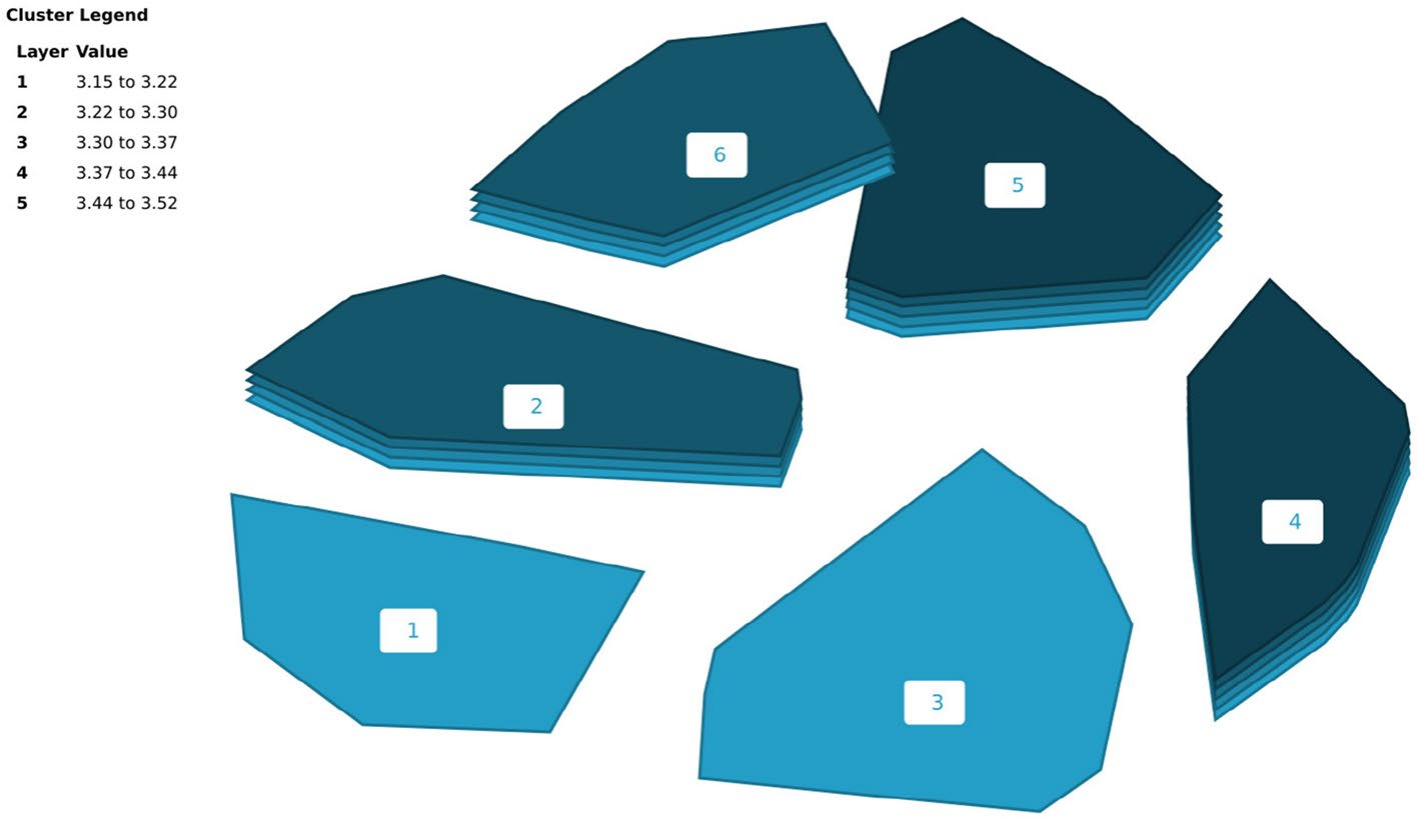
Cluster rating map

Results of the mean rating analysis showed that all six clusters were rated on average as moderately important (See Fig. 2). Cluster 5 (Collective Wisdom: Original Instructions and Dynamic Futures) had the highest importance ratings, followed by Cluster 4 (Intergenerational Healing and Flourishing), and Cluster 6 (Centering Indigenous Ways and Cultures in Research). Though there were small differences in mean rating values between clusters, it was apparent that participants felt that all clusters were important and represented Indigenous strengths-based health and wellness research.

## Discussion

This study responds to calls for the incorporation and definition of strengths-based Indigenous models of health and well-being, which are virtually non-existent within public health and medical research currently [3, 8, 19]. Indeed, only recently has colonization been acknowledged as a fundamental social determinant of health [35] despite decades of advocacy by Indigenous scholars to decolonize health research [4, 36].

This study revealed six clusters or thematic focus areas which may serve as the foundation for a framework of Indigenous strengths-based health research. Each cluster can be viewed as a lesson from Indigenous researchers on how to promote healing and flourishing across generations. These lessons build upon the collective wisdom of our past and current generations to center Indigenous knowledges and cultures in the research world. Indigenous knowledges and science have existed since the beginning of time and given our collective voices, visioning, passion, and commitment, we can work to decolonize research so Indigenous science exists, resists, and persists for generations to come. This work and the work our ancestors, Elders, families, and youth are transforming Indigenous futures while shifting societal narratives from deficit-based to strengths-based. To successfully move this work forward and make a positive impact on the fields of public health, however, Indigenous leadership is key. The Indigenous scholars participating and authoring this study suggest tangible, concrete orientations, and action steps to support Indigenous researchers and community partners in applying an Indigenous, strength-based focus in guiding future research and practice efforts (Table 2).

### Limitations and strengths

This investigation represents the perspectives of 27 Indigenous scholars and the small sample size does not reflect perspectives of all Indigenous researchers. Despite connection to several tribes and communities, Indigenous scholars recognize the dual roles and relationships that health researchers carry, especially those situated within Eurocentric academic institutions. Historically, Indigenous communities have suffered at the whims of non-Indigenous health researchers claiming to have the best intentions of the community, only to perpetrate harm on the communities they purport to serve [4, 36]. For these reasons, this study aimed to elicit feedback from Indigenous scholars who carry knowledges of their own communities without attempting to speak for any Indigenous community. How Indigenous communities would define an “Indigenous strengths-based approach to health and wellness research” is an empirical and localized question that requires separate engagement with communities. As a result, this investigation reflects the collective experience of a group of Indigenous scholars who embody Indigenous survivance [37] and who co-exist within the often-oppressive circumstances of Eurocentric academic institutions. There are also several strengths of this project. This research was designed and carried out by an all-Indigenous research team who centered their community and cultural values throughout the project. Further, group concept mapping is an inherently participatory research method and this project privileged Indigenous scholars’ voices and experiences, aligning with Indigenous Research Methodologies [22]. Finally, Indigenous scholars collectively shared positive experiences (e.g., connectedness to one another) from participating in this project during the virtual interpretation meetings, as well as via email communication to the GCM facilitation team throughout the entire project.

## Conclusions

Current approaches to public health research are inadequate to address widening health inequities [38] and the ubiquitous legacy of colonization on Indigenous community health [4]. This study reflects Indigenous scholars’ views on strengths-based research to rise above the limits of dominant orientations and methodologies and build upon critical Indigenous scholarship [36, 39]. Findings coalesce around six thematic clusters and represent a radical shift in orientation, approach, and priorities for health research that moves away from extraction towards relationality [40] and centers Indigenous communities and values. This Indigenous-led and -developed framework represents an important step forward for the field of Indigenous health research by offering actionable recommendations to foster research relationships that celebrate and build upon the strengths of communities to promote health and wellness at the individual, community, and population levels, and ultimately to partner in research that has measurable impact on health equity.

## Data Availability

The datasets generated and/or analyzed during the current study are not publicly available but are available from the corresponding author on reasonable request.

## Abbreviation

GCM: Group Concept Mapping
